# Supplementary material for: COZOID: contact zone identifier for visual analysis of protein-protein interactions
Source: BMC Bioinformatics. 2018 Apr 6;19:125. doi: 10.1186/s12859-018-2113-6 (PMC5889581; doi:10.1186/s12859-018-2113-6)
Supplement: Supplementary file 2 — Software build. Executable binary file of the software tool. (ZIP 118,784 kb) [file 12859_2018_2113_MOESM2_ESM.zip › cozoid/platform/docs/testng-6.5.1-javadoc.zip!/org/testng/ReporterConfig.html]

ReporterConfig (TestNG)


---


|  |  |  |
| --- | --- | --- |
| **Overview** \| **Package** \| **Class** \| **Tree** \| **Deprecated** \| **Index** \| **Help** | |  |
| **PREV CLASS**   **NEXT CLASS** | **FRAMES**    **NO FRAMES**     **All Classes** |  |
| SUMMARY: NESTED \| FIELD \| CONSTR \| METHOD | DETAIL: FIELD \| CONSTR \| METHOD |  |


---


## org.testng Class ReporterConfig

```
java.lang.Object
  org.testng.ReporterConfig
```

---

``` public class ReporterConfig extends java.lang.Object ```

Stores the information regarding the configuration of a pluggable report listener. Used also
in conjunction with the <reporter> sub-element of the Ant task
NOTE: this class needs to be public. It's used by TestNG Ant task

**Author:**
:   Cosmin Marginean, Apr 12, 2007

---

| **Nested Class Summary** | |
| --- | --- |
| `static class` | `ReporterConfig.Property` |


**Constructor Summary**

`ReporterConfig()`


| **Method Summary** | |
| --- | --- |
| `void` | `addProperty(ReporterConfig.Property property)` |
| `static ReporterConfig` | `deserialize(java.lang.String inputString)` |
| `java.lang.String` | `getClassName()` |
| `java.util.List<ReporterConfig.Property>` | `getProperties()` |
| `java.lang.Object` | `newReporterInstance()`             Creates a reporter based on the current configuration |
| `java.lang.String` | `serialize()` |
| `void` | `setClassName(java.lang.String className)` |
| `java.lang.String` | `toString()` |

**Methods inherited from class java.lang.Object**

`clone, equals, finalize, getClass, hashCode, notify, notifyAll, wait, wait, wait`

**Constructor Detail**

### ReporterConfig

```
public ReporterConfig()
```


**Method Detail**

### addProperty

```
public void addProperty(ReporterConfig.Property property)
```

---


### getProperties

```
public java.util.List<ReporterConfig.Property> getProperties()
```

---


### getClassName

```
public java.lang.String getClassName()
```

---


### setClassName

```
public void setClassName(java.lang.String className)
```

---


### serialize

```
public java.lang.String serialize()
```

---


### deserialize

```
public static ReporterConfig deserialize(java.lang.String inputString)
```

---


### newReporterInstance

```
public java.lang.Object newReporterInstance()
```

:   Creates a reporter based on the current configuration

---


### toString

```
public java.lang.String toString()
```

:   **Overrides:**: `toString` in class `java.lang.Object`


---


|  |  |  |
| --- | --- | --- |
| **Overview** \| **Package** \| **Class** \| **Tree** \| **Deprecated** \| **Index** \| **Help** | |  |
| **PREV CLASS**   **NEXT CLASS** | **FRAMES**    **NO FRAMES**     **All Classes** |  |
| SUMMARY: NESTED \| FIELD \| CONSTR \| METHOD | DETAIL: FIELD \| CONSTR \| METHOD |  |


---
